# Epidemiology of sepsis and septic shock in intensive care units between sepsis-2 and sepsis-3 populations: sepsis prognostication in intensive care unit and emergency room (SPICE-ICU)

**DOI:** 10.1186/s40560-020-00465-0

**Published:** 2020-06-30

**Authors:** Toshikazu Abe, Kazuma Yamakawa, Hiroshi Ogura, Shigeki Kushimoto, Daizoh Saitoh, Seitaro Fujishima, Yasuhiro Otomo, Joji Kotani, Yutaka Umemura, Yuichiro Sakamoto, Junichi Sasaki, Yasukazu Shiino, Naoshi Takeyama, Takehiko Tarui, Shin-ichiro Shiraishi, Ryosuke Tsuruta, Taka-aki Nakada, Toru Hifumi, Akiyoshi Hagiwara, Masashi Ueyama, Norio Yamashita, Tomohiko Masuno, Hiroto Ikeda, Akira Komori, Hiroki Iriyama, Satoshi Gando, for the JAAM SPICE Study Group

**Affiliations:** 1 Department of Health Services Research, Faculty of Medicine, University of Tsukuba, 1-1-1 Tennodai, Tsukuba, Ibaraki, 305-8577 Japan; 2 Health Services Research and Development Center, University of Tsukuba, Tsukuba, Japan; 3 Department of Emergency and Critical Care Medicine, Tsukuba Memorial Hospital, Tsukuba, Japan; 4 Division of Trauma and Surgical Critical Care, Osaka General Medical Center, Osaka, Japan; 5 Department of Traumatology and Acute Critical Medicine, Osaka University Graduate School of Medicine, Osaka, Japan; 6 Division of Emergency and Critical Care Medicine, Tohoku University Graduate School of Medicine, Sendai, Japan; 7 Division of Traumatology, Research Institute, National Defense Medical College, Tokorozawa, Japan; 8 Center for General Medicine Education, Keio University School of Medicine, Tokyo, Japan; 9 Trauma and Acute Critical Care Center, Medical Hospital, Tokyo Medical and Dental University, Tokyo, Japan; 10 Division of Disaster and Emergency Medicine, Department of Surgery Related, Kobe University Graduate School of Medicine, Kobe, Japan; 11 Emergency and Critical Care Medicine, Saga University Hospital, Saga, Japan; 12 Department of Emergency and Critical Care Medicine, Keio University School of Medicine, Tokyo, Japan; 13 Department of Acute Medicine, Kawasaki Medical School, Kurashiki, Japan; 14 Advanced Critical Care Center, Aichi Medical University Hospital, Nagakute, Japan; 15 Department of Trauma and Critical Care Medicine, Kyorin University School of Medicine, Tokyo, Japan; 16 Department of Emergency and Critical Care Medicine, Aizu Chuo Hospital, Aizuwakamatsu, Japan; 17 Advanced Medical Emergency & Critical Care Center, Yamaguchi University Hospital, Ube, Japan; 18 Department of Emergency and Critical Care Medicine, Chiba University Graduate School of Medicine, Chiba, Japan; 19 Department of Emergency and Critical Care Medicine, St. Luke’s International Hospital, Tokyo, Japan; 20 Department of Emergency Medicine, Niizashiki Chuo General Hospital, Niizashiki, Japan; 21 Department of Trauma, Critical Care Medicine, and Burn Center, Japan Community Healthcare Organization, Chukyo Hospital, Nagoya, Japan; 22 Advanced Emergency Medical Service Center, Kurume University Hospital, Kurume, Japan; 23 Department of Emergency and Critical Care Medicine, Nippon Medical School, Tokyo, Japan; 24 Department of Emergency Medicine, Teikyo University School of Medicine, Tokyo, Japan; 25 Department of General Medicine, Juntendo University, Tokyo, Japan; 26 Division of Acute and Critical Care Medicine, Hokkaido University Graduate School of Medicine, Sapporo, Japan; 27 Department of Acute and Critical Care Medicine, Sapporo Higashi Tokushukai Hospital, Sapporo, Japan

**Keywords:** Intensive care unit, Sepsis, In-hospital mortality

## Abstract

**Background:**

Diagnosing sepsis remains difficult because it is not a single disease but a syndrome with various pathogen- and host factor-associated symptoms. Sepsis-3 was established to improve risk stratification among patients with infection based on organ failures, but it has been still controversial compared with previous definitions. Therefore, we aimed to describe characteristics of patients who met sepsis-2 (severe sepsis) and sepsis-3 definitions.

**Methods:**

This was a multicenter, prospective cohort study conducted by 22 intensive care units (ICUs) in Japan. Adult patients (≥ 16 years) with newly suspected infection from December 2017 to May 2018 were included. Those without infection at final diagnosis were excluded. Patient’s characteristics and outcomes were described according to whether they met each definition or not.

**Results:**

In total, 618 patients with suspected infection were admitted to 22 ICUs during the study, of whom 530 (85.8%) met the sepsis-2 definition and 569 (92.1%) met the sepsis-3 definition. The two groups comprised different individuals, and 501 (81.1%) patients met both definitions. In-hospital mortality of study population was 19.1%. In-hospital mortality among patients with sepsis-2 and sepsis-3 patients was comparable (21.7% and 19.8%, respectively). Patients exclusively identified with sepsis-2 or sepsis-3 had a lower mortality (17.2% vs. 4.4%, respectively). No patients died if they did not meet any definitions. Patients who met sepsis-3 shock definition had higher in-hospital mortality than those who met sepsis-2 shock definition.

**Conclusions:**

Most patients with infection admitted to ICU meet sepsis-2 and sepsis-3 criteria. However, in-hospital mortality did not occur if patients did not meet any criteria. Better criteria might be developed by better selection and combination of elements in both definitions.

**Trial registration:**

UMIN000027452

## Key points

- The majority of patients with suspected infection admitted to the ICU met sepsis-2 and sepsis-3 definitions.
- In-hospital mortality did not occur if patients did not meet any sepsis definitions.

## Background

Sepsis is an aberrant or dysregulated host response resulting in organ dysfunctions and is different from infection [1]. It is not a single disease but a syndrome exhibiting with various symptoms caused by pathogens and host factors. Sepsis should be immediately recognized because it is the primary cause of death from infection, especially if not diagnosed and treated promptly. Sepsis-2 has high sensitivity [2] but captures mild infection and not infectious diseases. Sepsis-3 was established to improve risk stratification among patients with a suspected infection focusing on organ failures [1].

When considering previous studies about the diagnosis and taxonomy of sepsis to date [1, 3, 4], nearly all of them just defined sepsis as cases of high mortality due to infectious diseases. Sepsis studies may be controversial because they were unable to differentiate an aberrant or dysregulated host response itself from infection. The definitions of sepsis-2 and sepsis-3 have still been inadequate to accurately capture sepsis. Therefore, both definitions may have misclassified patients with sepsis as patients with infectious diseases. Although the true nature of sepsis remains to be identified, we should clearly know what the definitions of sepsis-2 and sepsis-3 indicate because different definitions could change its epidemiology to identify the clinical care, future research, and healthcare planning. Such information would facilitate the definition criteria of the next sepsis. Therefore, this study aimed to describe characteristics of patients who met sepsis-2 and sepsis-3 definitions.

## Methods

### Design and setting

This multicenter, prospective cohort study was conducted in an intensive care unit (ICU) subset of the Japanese Association for Acute Medicine Sepsis Prognostication in Intensive Care Unit and Emergency Room (JAAM SPICE-ICU), including 22 ICUs in Japan from December 2017 to May 2018.

### Participants

Adult patients (≥ 16 years) with newly suspected infection were included. Suspected infection was defined by the administration of any kind of antibiotic, and thereby a culture of body fluids or imaging should be conducted to identify the infectious pathogen. All patients were admitted to the ICUs in study hospitals. Exclusion criteria included patients who were not transferred from other hospitals and those without infection at the final diagnosis.

### Data collection

Data were extracted from the SPICE database, compiled by SPICE investigators. Collected variables included relevant patient information, such as demographics, comorbidities, degree of clinical frailty, vital signs, and site of infection. In-hospital mortality was identified as the primary outcome. Secondary outcomes were ventilator-free days (VFD), intensive care unit-free days (ICU-free days), length of hospital stay (LOS), and condition at discharge. Data collection was conducted as part of the clinical routine workup. SPICE site investigators recorded all data throughout the patient’s hospital stays. If case of missing data, the SPICE committee requested a reconfirmation of data extraction from SPICE investigators.

### Data definitions

Sepsis-2 was defined as having a suspected site of infection, ≥ 2 systemic inflammatory response syndrome criteria (SIRS) [5] and ≥ 1 organ dysfunction criteria [6]. Severe sepsis was actually defied as sepsis-2 according to the sepsis-2 definition [3]. Sepsis-3 was defined as having a suspected site of infection and organ dysfunction (an acute change in the total sequential organ failure assessment (SOFA) score of ≥ 2 points consequent to the infection) [1]. Regarding shock, sepsis-2 and sepsis-3 shocks were defined according to the sepsis-2 [6] and sepsis-3 definitions, respectively [1] (Supplemental file 1). Frailty was defined according to the Clinical Frailty Scale (CFS), an easy and intuitive determinable categorization tool based on simple visual descriptions [7]. Patients’ status for CFS before hospital admission was obtained from patients themselves or their relatives. Infection sites at final diagnosis included the lung, intra-abdominal, urinary tract, soft tissue, central nervous system (CNS), osteoarticular, endocardium, wound, catheter-related, implant device-related, others, or unidentified infections. The diagnosis of the infection site was recorded at discharge. Acute physiology and chronic health evaluation II (APACHE II) score was calculated at the initial examination instead of the worst data within 24 h. If APACHE II score was missing, zero was used instead of missing data. SOFA score was calculated similarly as the APACHE II score. VFD was defined as the number of days within the first 28 days post-admission that the patient can breathe without a ventilator. The VFD of patients who died during the study period was set as zero. ICU-free days were calculated similarly with VFD. Status at discharge was categorized as home, transfer to another facility (including long-term care and nursing homes), or death.

### Analysis

Patients with infection in ICUs were compared according to whether they met sepsis-2 or sepsis-3 definition. Patients were divided into five groups: sepsis-2; sepsis-3; sepsis-2 and sepsis-3; sepsis-2 and no sepsis-3; no sepsis-2 and sepsis-3; and no sepsis-2 and no sepsis-3. Descriptive statistics included proportions for categorical variables, and medians (interquartile range [IQR]) of continuous variables were calculated because not all variables were normally distributed. A few missing data was considered missing randomly. No assumptions were made on these data. All statistical analyses were performed using the Stata software version 15.1 (StataCorp, TX, USA).

## Results

**Fig. 1:**
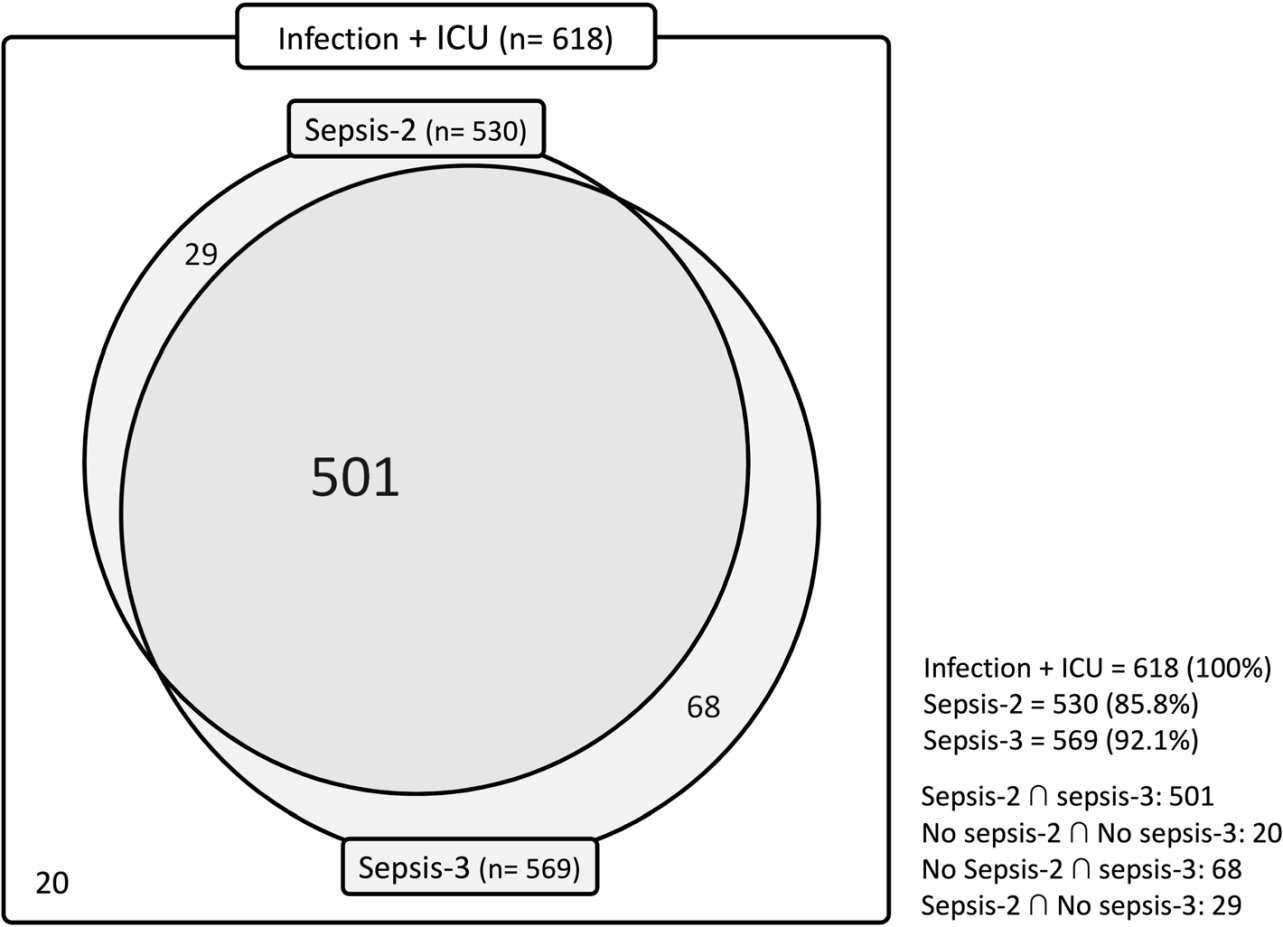
Taxonomy and in-hospital mortality among patients with infection admitted to the intensive care unit

A total of 618 patients with suspected infection admitted to 22 ICUs during the study period were included in this study. Among them, 530 (85.8%) patients had sepsis-2, and 569 (92.1%) had sepsis-3; most patients were overlapped; however, patients with sepsis-2 and sepsis-3 were different individuals. A total of 501 (81.1%) met both definitions, and 29 exclusively met the sepsis-2 (only sepsis-2), and 68 exclusively met the sepsis-3 (only sepsis-3) definition. A total of 20 patients did not meet either of the definitions (Fig. 1). Majority of patients were admitted to the ICUs directly from the emergency departments (EDs) (56.6%). A total of 592 (95.8%) patients were positive for SIRS, and 368 (59.6%) were positive for qSOFA. A total of 297 (48.1%) patients met the sepsis-2 shock criteria, and 109 (17.6%) met the sepsis-3 shock criteria.

Table 1 shows characteristics of patients with infection in ICUs according to sepsis definitions. The distributions of baseline characteristics such as age, sex, and comorbidities were comparable between sepsis-2 and sepsis-3. The baseline SOFA score for the sepsis-2 only group was 4 (IQR, 0–8) although the baseline SOFA for other groups was 0 or 1. Lactate and blood culture positivity were lower if they did not meet the definitions. The SOFA score was lower if they did not meet any definitions. The trend in pathogens and antibiotics according to sepsis definitions was also nonspecific; however, blood culture positivity was lower if patients did not meet any definitions (sepsis-2 or sepsis-3), and carbapenem was more frequently used in patients who met any definitions (Table 2).

**Table 1:** Case mix characteristics for admissions to ICU with suspected infection (by sepsis-2 and sepsis-3 definitions)

		Sepsis-2	Sepsis-3	Sepsis-2 and sepsis-3	Sepsis-2 and no sepsis-3	No sepsis-2 and sepsis-3	No sepsis-2 and no sepsis-3
Variables		*n* = 530	*n* = 569	*n* = 501	*n* = 29	*n* = 68	*n* = 20
Age at admission (year)		72 (60–81)	72 (60–81)	73 (61–81)	69 (57–75)	66 (57–81)	70 (55–79)
Gender (male)		299 (56.4)	318 (55.9)	281 (56.1)	18 (62.1)	37 (54.4)	13 (65.0)
BMI (kg/m^2^)		21.8 (19.4–24.3)	21.9 (19.3–24.3)	21.9 (19.5–24.3)	21.3 (19.3–24.2)	21.8 (18.3–24.2)	23.4 (19.2–26.3)
Admission source	Emergency department	304 (57.4)	317 (55.7)	285 (56.9)	19 (65.5)	32 (47.1)	14 (70.0)
	Non–ED (hospital/dept. transfers)	204 (38.5)	230 (40.4)	200 (39.9)	4 (13.8)	30 (44.1)	2 (10.0)
	Intensive care unit	22 (4.2)	22 (3.9)	16 (3.2)	6 (20.7)	6 (8.8)	4 (20.0)
Charlson comorbidity index	0	156 (29.4)	169 (29.7)	147 (29.3)	9 (31.0)	22 (32.3)	8 (40.0)
	1–2	227 (42.8)	249 (43.8)	214 (42.7)	13 (44.8)	35 (51.5)	8 (40.0)
	3–4	104 (19.6)	105 (18.5)	99 (19.8)	5 (17.2)	6 (8.8)	2 (10.0)
	> 4	43 (8.1)	46 (8.1)	41 (8.2)	2 (6.9)	5 (7.4)	2 (10.0)
Clinical Frailty Scale	Fit (CFS 1–3)	269 (50.9)	289 (50.8)	249 (49.8)	20 (71.4)	40 (58.8)	12 (60.0)
	Pre–frail (CFS 4)	90 (17.0)	100 (17.6)	89 (17.8)	1 (3.6)	11 (16.2)	4 (20.0)
	Frail (CFS 5–9)	169 (32.0)	179 (31.5)	162 (32.4)	87(25.0)	17 (25.0)	4 (20.0)
Immuno–insufficiency at APACHE II		110 (20.8)	111 (19.6)	101 (20.2)	9 (31.0)	10 (14.9)	3 (15.0)
Baseline SOFA for sepsis-3		0 (0–1)	0 (0–1)	0 (0–1)	4 (0–8)	0 (0–0)	1 (0–3)
Glasgow Coma Scale		12 (7–14)	12 (7–15)	11 (7–14)	14 (11–15)	14 (10–15)	15 (14–15)
Intubated		225 (42.6)	226 (39.7)	212 (42.3)	13 (44.8)	14 (20.6)	5 (26.3)
Systolic blood pressure (mmHg)		102 (82–126)	105 (84–128)	101 (82–125)	122 (107–143)	122 (107–141)	123 (111–143)
Heat rate (/min)		107 (90–124)	107 (89–122)	108 (91–124)	93 (82–108)	90 (80–108)	90 (79–106)
Respiratory rate (/min)		24 (19–30)	24 (19–30)	24 (19–30)	19 (17–24)	20 (18–26)	20 (19–25)
Body temperature (°C)		37.3 (36.5–38.5)	37.4 (36.5–38.4)	37.4 (36.5–38.5)	37.1 (36.3–38.5)	37.5 (36.8–38.3)	37.7 (36.7–38.3)
Lactate (mmol/L)		2.9 (1.7–4.9)	2.7 (1.5–4.7)	3.1 (1.7–5.1)	2.1 (1.4–2.9)	1.2 (0.9–1.8)	1.0 (0.9–1.6)
Positive blood cultures		252 (50.0)	261 (48.4)	245 (51.3)	7 (26.9)	16 (23.5)	4 (23.5)
Site of infection	Lung	200 (37.7)	208 (36.6)	192 (38.3)	8 (27.6)	16 (23.5)	8 (40.0)
	Abdomen	111 (20.9)	119 (20.9)	101 (20.2)	10 (34.5)	18 (26.5)	2 (10.0)
	Urinary tract	91 (17.2)	101 (17.8)	89 (17.8)	2 (6.9)	12 (17.7)	3 (15.0)
	Soft tissue	65 (12.3)	70 (12.3)	60 (12.0)	5 (17.2)	10 (14.7)	6 (30.0)
	Central nervous system	11 (2.1)	13 (2.3)	10 (2.0)	1 (3.5)	3 (4.4)	1 (5.0)
	Intravenous catheter	4 (0.8)	4 (0.7)	4 (0.8)	0	0	0
	Osteoarticular	4 (0.8)	6 (1.1)	4 (0.8)	0	2 (2.9)	0
	Endocardium	1 (0.2)	1 (0.2)	1 (0.2)	0	0	0
	Wound	5 (0.9)	5 (0.9)	4 (0.8)	1 (3.5)	1 (1.5)	0
	Implant device	2 (0.4)	2 (0.4)	2 (0.4)	0	0	0
	Other	14 (2.6)	16 (2.8)	13 (2.6)	1 (3.5)	3 (4.4)	0
	Unidentified	22 (4.2)	24 (4.2)	21 (4.2)	1 (3.5)	3 (4.4)	0
Organ dysfunction on arrival	Acute lung injury with pneumonia	127 (24.0)	124 (21.8)	124 (24.8)	3 (10.3)	0	0
	Acute lung injury without pneumonia	96 (18.2)	99 (17.5)	94 (18.8)	2 (7.1)	5 (7.4)	0
	ARDS (Berlin criteria)	57 (10.9)	60 (10.7)	56 (11.3)	1 (3.6)	4 (6.0)	0
	Mechanical ventilation use	227 (43.0)	230 (40.6)	215 (43.1)	12 (41.4)	15 (22.1)	4 (22.2)
	Urine output (ml/24 h)	896 (404–1488)	910 (407–1503)	873 (395–1440)	1220 (787–1767)	1147 (723–1770)	1300 (930–1830)
	Oliguria	238 (45.1)	231 (40.8)	226 (45.3)	12 (41.4)	5 (7.4)	3 (15.0)
	Acute kidney injury in APACHE II	257 (48.7)	263 (46.4)	251 (50.3)	6 (20.7)	12 (17.7)	0
	Sepsis-2 shock criteria	297 (56.0)	288 (50.6)	288 (57.5)	9 (31.0)	0	0
	Sepsis-3 shock criteria	108 (20.4)	107 (18.8)	106 (21.2)	2 (6.9)	1 (1.5)	0
SIRS ≥ 2		530 (100)	549 (96.5)	501 (100)	29 (100)	48 (70.6)	14 (70.0)
qSOFA ≥ 2		345 (65.1)	356 (62.6)	336 (67.1)	9 (31.0)	20 (29.4)	3 (15.0)
SOFA score		8 (5–11)	8 (5–11)	8 (6–11)	5 (1–8)	4 (3–5)	1 (1–2)
APACHE II score		21 (15–28)	20 (15–27)	21 (16–28)	13 (10–18)	14 (10–17)	11 (7–14)

Reported counts (proportions) for categorical variables and median (interquartile range) for continuous variables

Missing; BMI = 5; Clinical Frailty Scale = 2; Immuno-insufficiency at APACHE II = 3; Intubation = 1; Systolic blood pressure = 2; Heart rate = 1; Body temperature = 1; Lactate = 14; Blood culture = 36; Acute lung injury without pneumonia = 3; ARDS = 9; Mechanical ventilation use = 4; Urine output = 36; Oliguria = 2; Acute kidney injury at APACHE II = 2

*BMI* body mass index, *ED* emergency department, *ICU* intensive care unit, *CFS* Clinical Frailty Scale, *APACHE II* Acute physiology and chronic health evaluation II, *SOFA* sequential (sepsis–related) organ failure assessment, *WBC* white blood cell, *ARDS* acute respiratory distress syndrome, *SIRS* systemic inflammatory response syndrome

**Table 2:** Pathogens and antibiotics according to the sepsis definitions

		Sepsis-2	Sepsis-3	Sepsis-2 and sepsis-3	Sepsis-2 and no sepsis-3	No sepsis-2 and sepsis-3	No sepsis-2 and no sepsis-3
Variables		*n* = 530	*n* = 569	*n* = 501	*n* = 29	*n* = 68	*n* = 20
Microbiology of blood cultures							
Gram-negative	E. coli	84 (15.9)	89 (15.6)	84 (16.8)	0	5 (7.4)	0
	Klebsiella	35 (6.6)	34 (6.0)	34 (6.8)	1 (3.5)	0	1 (5.0)
	Pseudomonas	2 (0.4)	2 (0.4)	2 (0.4)	0	0	0
Gram-positive	Staphylococci	62 (11.7)	66 (11.6)	60 (12.0)	2 (6.9)	6 (8.8)	0
	Streptococci	43 (8.1)	45 (7.9)	42 (8.4)	1 (3.5)	3 (4.4)	1 (5.0)
	MRSA	5 (0.9)	6 (1.1)	5 (1.0)	0	1 (1.5)	0
	Enterococcus	12 (2.3)	12 (2.1)	12 (2.4)	0	0	0
	Anaerobic	14 (2.6)	12 (2.1)	12 (2.4)	2 (6.9)	0	0
Fungi		2 (0.4)	2 (0.4)	2 (0.4)	0	0	0
Antibiotics							
Penicillin derivative (PCG, ABPC, ABPC/MCIPC)		21 (4.0)	27 (4.8)	21 (4.2)	0	6 (8.8)	1 (5.0)
Ampicillin/sulbactam		69 (13.0)	77 (13.5)	66 (13.2)	3 (10.3)	11 (16.2)	7 (35.0)
PIPC/TAZ		73 (13.8)	73 (12.8)	64 (12.8)	9 (31.0)	9 (13.2)	6 (30.0)
First generation cephalosporin		11 (2.1)	11 (2.0)	9 (1.8)	2 (6.9)	2 (2.9)	3 (15.0)
Second generation cephalosporin (CTM, CMZ, FMOX)		13 (2.5)	17 (3.0)	11 (2.2)	2 (6.9)	6 (8.8)	0
Third generation cephalosporin (CTX, CPZ, CTRX)		60 (11.3)	67 (11.8)	57 (11.4)	3 (10.3)	10 (14.7)	1 (5.0)
Third generation cephalosporin against pseudomonas		3 (0.6)	3 (0.5)	3 (0.6)	0	0	0
Fourth generation cephalosporin against pseudomonas		30 (5.7)	29 (5.1)	29 (5.8)	1 (3.5)	0	0
Carbapenem		279 (52.6)	291 (51.1)	268 (53.5)	11 (37.9)	23 (33.8)	3 (15.0)
Aminoglycoside		0	0	0	0	0	0
Quinolone		32 (6.0)	35 (6.2)	32 (6.4)	0	3 (4.4)	0
Tetracycline		3 (0.6)	3 (0.5)	3 (0.6)	0	0	0
Macrolide		29 (5.5)	29 (5.1)	29 (5.8)	0	0	0
Metronidazole		18 (3.4)	18 (3.2)	18 (3.6)	0	0	0
CLDM		23 (4.3)	24 (4.2)	20 (4.0)	3 (10.3)	4 (5.9)	1 (5.0)
Vancomycin		81 (15.3)	80 (14.1)	75 (15.0)	6 (20.7)	5 (7.4)	4 (20.0)
Other anti–methicillin-resistant staphylococcus aureus drugs		45 (8.5)	48 (8.4)	45 (9.0)	0	3 (4.4)	0
Antifungus		28 (5.3)	30 (5.3)	28 (5.6)	0	2 (2.9)	1 (5.0)
Others (ST, CLL, FOM, SBT/CPZ, AMPC)		10 (1.9)	11 (1.9)	10 (2.0)	0	1 (1.5)	1 (5.0)

Reported counts (proportions)

*ICU* intensive care unit, *PCG* penicillin G, *ABPC* ampicillin, *ABPC/MCIPC* ampicillin/cloxacillin, *PIPC/TAZ* tazobactam/piperacillin, *CTM* cefotiam, *CMZ* cefmetazole, *FMOX* flomoxef, *CTX* cefotaxime, *CPZ* cefoperazone, *CTRX* ceftriaxone, *CLDM* clindamycin, *ST* sulfamethoxazole-trimethoprim, *CLL* cefaclor, *FOM* fosfomycin, *SBT/CPZ* sulbactam/cefoperazone, *AMPC* amoxicillin, *MRSA* methicillin-resistant Staphylococcus aureus

In-hospital mortality of study population was 19.1%. In-hospital mortality among patients with sepsis-2 and sepsis-3 patients was comparable (21.7% and 19.8%, respectively) (Table 3). Patients exclusively identified by sepsis-2 or sepsis-3 had a lower mortality (17.2% vs. 4.4%, respectively). No patients died if they did not meet any definitions. Patients who met sepsis-3 shock criteria had higher in-hospital mortality than those who met sepsis-2 shock criteria.

**Table 3:** Outcomes according to the sepsis definitions

		Sepsis-2	Sepsis-3	Sepsis-2 and sepsis-3	Sepsis-2 and no sepsis-3	No sepsis-2 and sepsis-3	No sepsis-2 and no sepsis-3
Outcomes		*n* = 530	*n* = 569	*n* = 501	*n* = 29	*n* = 68	*n* = 20
In-hospital mortality	All	114/526 (21.7)	112/565 (19.8)	109/497 (21.9)	5/29 (17.2)	3/68 (4.4)	0/19 (0)
	Shock Sepsis-2 Criteria	80/295 (27.1)	78/286 (27.3)	78/286 (27.3)	2/9 (22.2)	0	0
	Shock Sepsis-3 Criteria	36/107 (33.6)	36/106 (34.0)	36/105 (34.3)	0/2 (0)	0/1 (0)	0
	Patients from ED	57/303 (18.8)	55/316 (17.4)	54/284 (19.0)	3/19 (15.8)	1/32 (3.1)	0/13 (0)
Survivor dispositions	Home	152/526 (28.9)	171/565 (30.3)	140/497 (28.2)	12/29 (41.4)	31/68 (45.6)	10/19 (52.6)
	Transfer	260/526 (49.4)	282/565 (49.9)	248/497 (49.9)	12/29 (41.4)	34/68 (50.0)	9/19 (47.4)
28-day mortality		98/503 (19.5)	99/536 (18.5)	96/474 (20.3)	2/29 (6.9)	3/62 (4.8)	0/19 (0)
ICU-free days		13 (0–21)	15 (0–21)	13 (0–21)	19 (2–24)	22 (15–25)	24 (23–26)
Ventilator-free days		20 (0–28)	21 (0–28)	20 (0–26)	21 (0–28)	27 (22–28)	28 (28–28)
Length of hospital stay		25 (11–46)	24 (12–45)	25 (11–46)	31 (12–47)	20 (12–41)	11 (5–25)

Reported counts (proportions) for categorical and median (interquartile range) for continuous variables

Missing: In-hospital mortality = 5; 28-day mortality = 34; ICU-free days = 34; Ventilator-free days = 34; Length of hospital stay = 5

*ICU* intensive care unit, *ED* emergency department

## Discussion

### Summary

Characteristics and in-hospital mortality were compared according to sepsis-2 and sepsis-3 definitions in this prospective observational cohort of ICU patients. Almost all patients presenting to ICU with infection fulfill both definitions, but what each definition identified is different. However, in-hospital mortality was zero if patients did not meet any definitions. Better criteria might be developed by better selection and combination of elements in both definitions.

### Comparison with previous studies

Nearly all patients admitted to ICU with suspected infection fulfill both definitions in our study as well as in previous studies [8, 9]. In a retrospective cohort study of ICUs in England, with similar setting as in our study, sepsis-2 and sepsis-3 definitions identified similar populations (92% overlapped), which is consistent with that of our study (81% overlapped). Actually, 95% of patients overlapped if sepsis-3 definition was evaluated in sepsis-2 (severe sepsis) population in our cohort. Their severity scores, such as the SOFA, were derived from an estimation such as a receipt of organ support and could have been over- or underestimated in the study [9]. Another study also virtually calculated the SOFA score, even though it is one of the most important elements in sepsis-3 definition [8]. Previous studies reported some variations of epidemiology by data sources, data acquisition timing, and interpretation of organ failure criteria in sepsis criteria [8, 10, 11]. Our prospective study was designed to compare sepsis-2 and sepsis-3 among patients with suspected infection and directly confirmed results of previous studies [8, 9]. However, these minor variations in the precise interpretation of definitions may have not affect characteristic and mortality differences, especially in the ICU setting [8].

Patients exclusively identified with sepsis-2 or sepsis-3 had different characteristics when compared to patients with both sepsis-2 and sepsis-3. Although a total of 63% patients were diagnosed with sepsis-3 using qSOFA, only 29% of patients with sepsis-2(−)/sepsis-3(+) were diagnosed with sepsis-3 using qSOFA. Patients exclusively identified with sepsis-2 or sepsis-3 presumably included those who have clinically unmeasured features such as vague symptoms [12]. Patients who meet only one definition may need more attention because their symptoms were not prominent. Since sepsis-2 captures high level of inflammation, a patient with sepsis-2 would still need further attention; however, a negative sepsis-3 does not meet the current definition of sepsis. In-hospital mortality differed by approximately four times between the sepsis-2(+)/sepsis-3(−) group and the sepsis-2(−)/sepsis-3(+) group (17% vs. 4%). Although the number of patients was small, judging by one definition alone may cause misclassification of poor outcome patients who may be identified by the other definition. However, since they were actually in the ICU, the physician did not misclassify the patients. This highlights the limitations of both the definitions.

### Possible explanations and implications

Since sepsis-2 does not include an increased acute SOFA score from the baseline, any chronic organ failure may possibly be regarded as an acute organ failure. SOFA score was not identified even though sepsis-2 was defined based on this score, except for chronic organ failures. In our cohort, 96 (16%) patients had “not available” (NA) sepsis-3 baseline SOFA, which was indicated as zero according to the sepsis-3 definition, although all data of chronic organ failures were tried to obtain. Therefore, a number of patients with unknown chronic organ failures at baseline should have been included in those with acute organ failure in any definitions. Either way, the sepsis-3 definition has become more clinically objectively understandable than the sepsis-2 definition. Moreover, the sepsis-3 definition was originally easier to evaluate than the sepsis-2 definition.

Excluding SIRS as the starting point for sepsis-3 did not affect the incidence as majority of patients with organ failures also tend to have SIRS. The sepsis-3 shock was associated with a higher risk of death than sepsis-2 [13], because the sepsis-3 shock requires the presence of elevated serum lactate levels in addition to fluid-resistant hypotension [14]. The problem of sepsis diagnosis has been a little arbitrary, with differences in epidemiology. The sepsis-3 definition may be advantageous because it may increase the comparability of sepsis incidence and related mortality among studies by possibly reducing the subjective interpretation [8]. A consistent diagnosis of sepsis and septic shock between institutions should be considered not only for research purposes but also for quality measurement.

Generally, severity scores such as SOFA were better used for clinical research and quality measurement rather than risk assessment. Therefore, the definition of sepsis has undoubtedly dramatically advanced the research due to enhanced medical research efficiency when agreed disease and outcome definitions are used. However, definitions are still insufficient and have not been used beyond as tools of research and quality measurement.

When a patient was admitted to ICU due to an infection, his or her mortality rate was approximately 20% in this cohort. This will make little contribution even if new criteria are used because 20% is one of the highest mortality in ICU diseases. The advancement of sepsis definitions may lead to the concept that similar conditions were caused by infections, despite the different backgrounds and triggers. However, risk stratifications and predictions should be investigated in detail in the future. For example, the upgrade or downgrade type of sepsis should be assessed based on the immune response, a subgroup for site of infection, or a phenotype of treatment responsiveness. “One size fits all approach” has reached its limits.

### Limitations

This study has several limitations. First, organ failure data before the ICU admission were missing in some patients, which was also noticed in the original sepsis-3 study [1]. Second, regarding to APACHE II score, data at initial diagnosis were used instead of the worst data within 24 h of ICU admission because of availability. This may have led to underestimation of the severity of patient conditions. Third, missing data were indicated as zero in the APACHE II and SOFA scores, if some elements were missing. This would have been used to identify any underestimation of the variance of patient’s severity. However, effects of missing data should be small because missing data of elements were few.

## Conclusions

A majority of the patients who were admitted to the ICU with suspected infection met sepsis-2 and sepsis-3 definitions. In-hospital mortality was indicated as zero if patients did not meet any sepsis definitions.

## Supplementary information

**Additional file 1:.** Supplemental file 1.

## Data Availability

The datasets generated during and/or analyzed during the current study are available from the corresponding author on reasonable request.

## Abbreviations

SPICE-ICU: Sepsis Prognostication in Intensive Care Unit and Emergency Room
ICUs: Intensive care units
VFD: Ventilator-free days
ICU-free days: Intensive care unit-free days
LOS: Length of hospital stay
SIRS: Systemic inflammatory response syndrome criteria
SOFA: Sequential organ failure assessment
CFS: Clinical Frailty Scale
CNS: Central nervous system
APACHE II: Acute physiology and chronic health evaluation II
ED: Emergency department
NA: Not available
