# Supplementary material for: Epidemiology of sepsis and septic shock in intensive care units between sepsis-2 and sepsis-3 populations: sepsis prognostication in intensive care unit and emergency room (SPICE-ICU)
Source: J Intensive Care. 2020 Jun 30;8:44. doi: 10.1186/s40560-020-00465-0 (PMC7324770; doi:10.1186/s40560-020-00465-0)
Supplement: Supplementary file 1 — Additional file 1:. Supplemental file 1. [file 40560_2020_465_MOESM1_ESM.docx]

Sepsis-2 (severe sepsis) definition

A subject had to have a suspected site of infection, ≥ 2 systemic inflammatory response syndrome criteria, and ≥1 organ dysfunction criteria.

| Systemic inflammatory response syndrome criteria (SIRS) |
| --- |
| Fever ( > 38.3°C) or Hypothermia (core temperature < 36°C) |
| Altered mental status |
| Heart rate > 90/min |
| Leukocytosis (WBC count > 12,000/μL), Leukopenia (WBC count < 4000/μL), or Normal WBC count with greater than 10% immature forms |
| Tachypnea ( ≧20/min), PaCO2 ≦ 32 Torr, on mechanical ventilation |
| Hyperglycemia (plasma glucose > 140mg/dL) in the absence of diabetes |

Adapted from ^1^

| Organ dysfunction criteria (except chronic organ failure) |
| --- |
| Systolic blood pressure (SBP) < 90 mm Hg, or mean arterial pressure (MAP) < 65 mm Hg |
| an SBP decrease > 40mm Hg from patient’s baseline SBP |
| Creatinine > 2.0mg/dL |
| Acute oliguria (urine output < 0.5 mL/kg/hr for at least 2 hrs) |
| Hyperbilirubinemia (plasma total bilirubin > 2 mg/dL) |
| Thrombocytopenia (platelet count < 100,000/μL) |
| Hyperlactatemia ( > 2 mmol/L or 18.0mg/dL) |
| Coagulopathy (international normalized ratio > 1.5) |
| Acute lung injury with PaO2/FIO2 < 250 in the absence of pneumonia as infection source |
| Acute lung injury with PaO2/FIO2 < 200 in the presence of pneumonia as infection source |

Adapted from ^2^

Sepsis-3 definition^3^

A subject had to have a suspected site of infection such as qSOFA ≥ 2, and organ dysfunction can be identified as an acute change in total SOFA score　≥　2 points consequent to the infection. The baseline Sequential [Sepsis-related] Organ Failure Assessment (SOFA) score was assumed to be zero unless the patient is known to have preexisting (acute or chronic) organ dysfunction before the onset of infection.

| Sepsis-2 shock |
| --- |
| SBP < 90 mmHg |
| MBP < 65 mmHg |
| an SBP decrease > 40mm Hg |

A subject had to have ≥1 sepsis-2 shock despite adequate volume resuscitation.

Adapted from ^4^

| Sepsis-3 shock^3^ |
| --- |
| Adequate volume resuscitation |
| Requiring vasopressors to maintain MAP > 65 mm Hg |
| Serum lactate level >2 mmol/L (18 mg/dL) |

A subject had to have all of sepsis-3 shock criteria.

Reference

1. Levy MM, Dellinger RP, Townsend SR, et al. The Surviving Sepsis Campaign: results of an international guideline-based performance improvement program targeting severe sepsis. *Crit Care Med.* 2010;38(2):367-374.

2. Dellinger RP, Levy MM, Rhodes A, et al. Surviving sepsis campaign: international guidelines for management of severe sepsis and septic shock: 2012. *Crit Care Med.* 2013;41(2):580-637.

3. Singer M, Deutschman CS, Seymour CW, et al. The Third International Consensus Definitions for Sepsis and Septic Shock (Sepsis-3). *JAMA.* 2016;315(8):801-810.

4. Levy MM, Fink MP, Marshall JC, et al. 2001 SCCM/ESICM/ACCP/ATS/SIS International Sepsis Definitions Conference. *Crit Care Med.* 2003;31(4):1250-1256.
